# Efficacy and safety of ketogenic diet in glioblastoma: an updated systematic review and meta-analysis

**DOI:** 10.1007/s10072-026-09035-y

**Published:** 2026-04-25

**Authors:** Jawaria Firdous, Abdul Eizad Asif, Hafiz Muhammad Haris, Uzair Iqbal, Muddassir Khalid, Hafiza Fatima Zahid, Moosa Mubarika, Iman Osman Abufatima, Mohammed Hammad Jaber Amin, Sadia Hafeez, Farha Jabeen, Muhammad Shahzaib, Noor Fatima, Nikil Kumar, Shuvendu Sen, Anid Hassan

**Affiliations:** 1 Department of Medicine, Hackensack Meridian Health Jersey Shore University Medical Center, Neptune City, NJ 07753 USA; 2 Punjab Medical College, Punjab, Pakistan; 3 Shalamar Medical and Dental College: Shalamar Medical and Dental College, Lahore, Pakistan; 4 King Edward Medical College: King Edward Medical University, Lahore, Pakistan; 5 Yale New Haven Hospital, New Haven, 06510 USA; 6 Nishtar Medical University, Multan, Pakistan; 7 University of Medical Sciences and Technology, Khartoum, Sudan; 8 Alzaiem Alazhari University, Khartoum North, Sudan; 9 Peoples University of Medical and Health Sciences for Women, Nawabshah, Pakistan; 10 Liaquat University of Medical and Health Sciences, Jamshoro, Pakistan

**Keywords:** Glioma, Glioblastoma, Ketogenic diet, Ketosis, Fasting

## Abstract

**Purpose:**

Glioblastoma (GBM) remains the most common and lethal primary central nervous system tumor, with a median survival of only 14.6 months under standard care. The tumor’s characteristic "Warburg effect"—a dependency on aerobic glycolysis for energy—creates a metabolic vulnerability. This review evaluates the efficacy and safety of the ketogenic diet (KD) as an adjunctive metabolic therapy aimed at exploiting this glucose dependency.

**Methods:**

This PRISMA-compliant systematic review updates clinical evidence by synthesizing data from databases from 2000 to September 2025. We searched PubMed, Embase, Cochrane, and Web of science for human studies assessing the ketogenic diet (KD) in glioblastoma and high-grade gliomas. Primary outcomes included overall survival (OS), progression-free survival (PFS), feasibility, and adverse events. Study quality was assessed using Joanna Briggs Institute tools. Prospero registration: CRD420251232650.

**Results:**

Forty-one studies were included, ranging from randomized trials to case series and abstracts, utilizing interventions such as the classic 4:1 ketogenic diet, Modified Atkins Diet, and calorie restriction. Adherence was high (> 75% maintained nutritional ketosis). Recent data indicate significant survival benefits; adherent cohorts achieved a median OS of 29.4 months vs 14.6 months in historical controls; with 66.7% 3-year survival rate. The diet was well-tolerated, with adverse events limited to mild gastrointestinal symptoms and fatigue. No Grade 3/4 diet-related toxicities reported.

**Conclusion:**

Current evidence supports the KD as a safe, feasible, and biologically rational adjunct to standard glioblastoma treatment. It demonstrates potential to prolong survival without severe toxicity, highlighting the need for standardized Phase III trials to establish clinical guidelines.

**Supplementary Information:**

The online version contains supplementary material available at 10.1007/s10072-026-09035-y.

## Introduction

GBM, the most common malignant CNS tumor, accounts for more than half of malignant CNS tumors with US age-adjusted incidence of 3.27 per 100,000 and global rate of 3–5/100,000 [1, 2]. Despite Stupp protocol (maximum safe resection + radiotherapy + temozolomide), median survival remains 14.6 months with 2-year survival of 26.5% [3]. Older age, incomplete resection, and unmethylated MGMT predict poor outcomes [4].

A major reason for treatment resistance in GBM is abnormal metabolic behavior. GBM exhibits the ‘‘Warburg effect’’-aerobic glycolysis despite oxygen availability-creating glucose addiction via PKM2 activation, HIF–1α upregulation, and glutaminolysis that fuels proliferation, and therapy resistance [5]. This metabolic vulnerability- creates a therapeutic opportunity for KD, which mimic metabolic state of fasting by lowering glucose availability to tumor cells while providing ketones as alternative fuel for healthy neurons [6].

However, KD’s clinical role in GBM remains uncertain. Variations in diet formulation, targets for ketosis, duration of the intervention, macronutrient ratios, and timing relating to standard interventions make study comparison difficult. Patient selection and disease stage further complicate interpretation. Thus, ketogenic diets are not integrated into standard GBM guidelines. Evidence is limited by small cohorts (*n* = 6–29), heterogeneous designs, and the absence of any phase III trials [6, 7].

The classical KD that uses a 4:1 ratio of fat to combined carbohydrate and protein, induces a state of systemic ketosis (β-hydroxybutyrate greater than 0.5 mmol/L), reduced blood glucose and insulin levels, downregulation of the Akt/mTOR signalling pathway, decreases glycolysis by reduced activity of PKM2 and GLUT1. Alongside, the ketogenic diet also promotes the apoptosis through activation of AMPK. Preclinical GBM models demonstrate tumor growth reduction, radiosensitization, HIF-1α downregulation, and chemotherapy synergy with ketogenic diet [6].

Phase I/II trial demonstrates robust feasibility (> 79% compliance, > 50% ketosis days alongside Stupp), mild adverse effects(nausea, fatigue, zero grade ≥ 3) and preserved quality of life [7]. Adherent cohorts achieve median PFS 12.9 months (vs 6.7 months historical) and OS 29.4 months (vs 14.6 months) [7], with 66.7% vs 8.3% 3-year survival [8]. Duraj et al. illustrates the application of GKI-monitored KD as a form of ketogenic metabolic therapy for targeting glycolysis/glutaminolysis in cancer cells by substrate competition with ineffective ketones for cell growth [9].

Notably, the 2024 systematic review of Valerio et al. excluded pre-2005 studies and all trials/publications in 2025, creating an evidence gap [6]. This PRISMA-compliant systematic review updates the 2024 analysis by pooling all clinical trials and original research on GBM/glioma since 2000, including pioneering pre-2005 trials and ongoing 2025 investigations. We synthesize current clinical evidence to assess how ketogenic diets influence feasibility, survival outcomes (PFS/OS), adverse effects, and quality of life in patients with GBM/glioma across all disease stages alone or in combination with standard/nonconventional therapies.

## Methods

This systematic review and meta-analysis were conducted per Cochrane standards [10] and the PRISMA guidelines [11]. Prospero registration: CRD420251232650.

### Literature search

Databases to be searched: PubMed, Web of Science, Embase and Cochrane from 2000 to September 2025. Search architecture constructed using MeSH/Emtree headings and keyword permutations. Boolean structure deployed: (“ketogenic diet” AND “glioblastoma”). Detailed search strings for the aforementioned databases are available in the (Supplementary Table 2).

Inclusion criteria consist of peer-reviewed human studies, including randomized designs, cohorts, case reports, case series, or mixed-method clinical evaluations; ketogenic or carbohydrate-restricted dietary interventions administered with measurable adherence; and explicit reporting of survival, progression, radiologic response, metabolic outcomes, quality-of-life metrics, or nutritional feasibility indicators. Exclusion criteria consist of preclinical studies, narrative reviews, interventions that did not meet ketogenic thresholds, incomplete outcome reporting, and duplicate populations across publications.

Two independent reviewers screened titles/abstracts and full texts; a third reviewer resolved discrepancies. PRISMA flow mapping was prepared to document identification, screening, eligibility, and inclusion counts.

### Data extraction

Extracted data included: bibliographic identifiers (Study ID, location); study design (randomized, cohort, case series); sample size; demographic characteristics; age (year), diagnostic criteria for glioblastoma or high-grade glioma. Interventions recorded the type of ketogenic diet, duration of dietary exposure(months, range), and follow-up time(months, range). Clinical endpoints included overall survival, progression-free survival, and Adverse events related to the ketogenic diet. Extraction was performed independently by two reviewers. All entries were cross-checked for accuracy, and discordances were reconciled through joint review. Authors were contacted when necessary for missing numerical data or unclear intervention details.

## Results

### Process of study selection

**Fig. 1:**
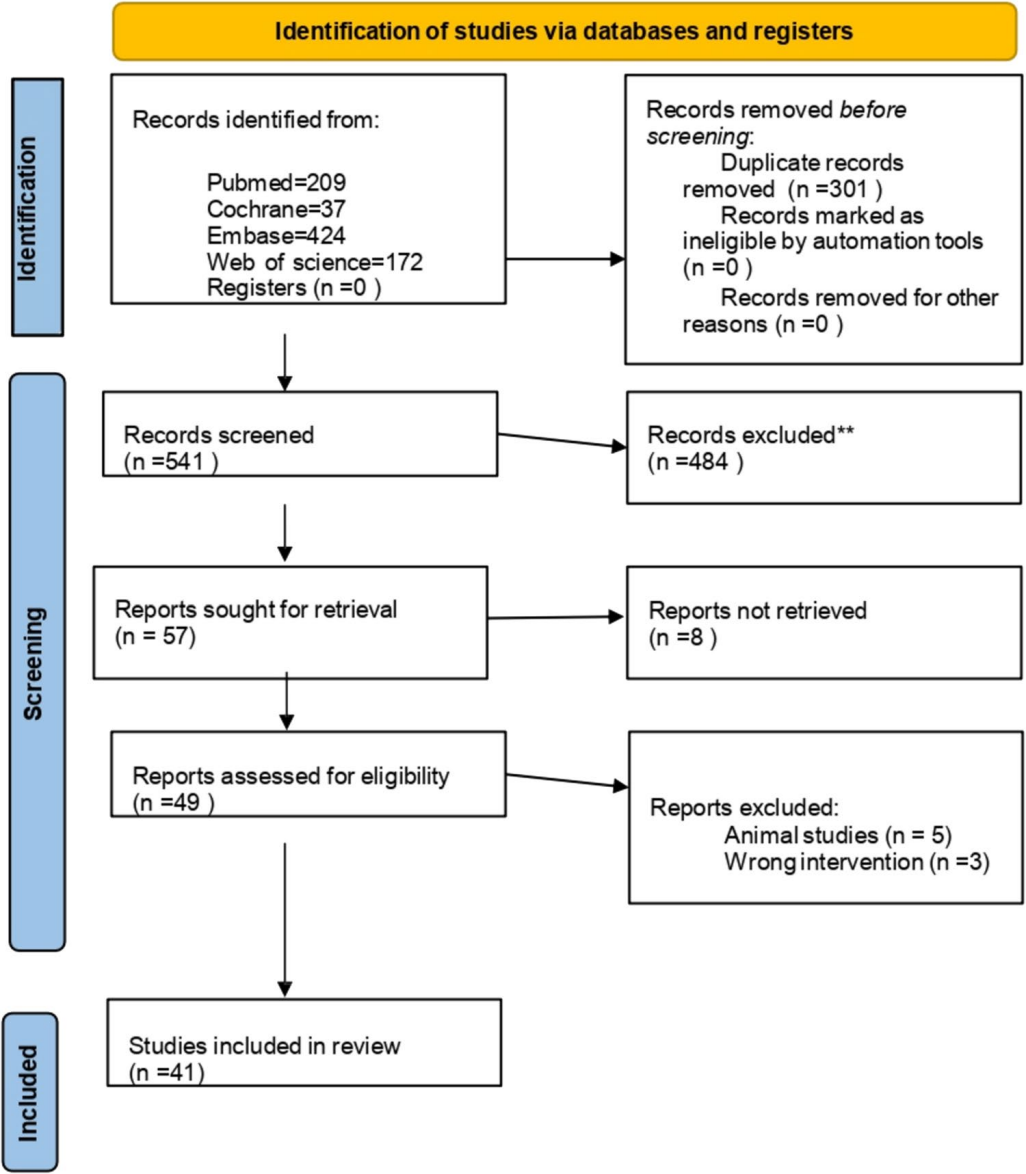
PRISMA 2020 flow diagram for new systematic reviews included searches of databases and registers only

Database searches resulted 1,039 articles: PubMed (*n* = 206), Cochrane (*n* = 237), Embase (*n* = 424), and Web of Science (*n* = 172). After removing 301 duplicates, 541 records underwent screening. Following full text screening, 484 records were excluded, leaving 57 reports sought for retrieval. Eight reports could not be obtained. Of 49 full-text reports assessed, 8 were excluded (5 animal studies, 3 non-ketogenic interventions). Ultimately, 41 studies met inclusion criteria [7, 8, 12–42]. We included 8 abstracts as well. The PRISMA flow diagram is shown in (Fig. 1).

### Characteristics of included studies

A total of 41 published studies were included, comprising a mix of prospective and retrospective cohorts, clinical trials, case series, and case reports, with most conducted in the United States, Europe, and Asia. Study sample sizes ranged from single-patient reports to cohorts of over 100 patients, primarily focusing on glioblastoma multiforme (GBM) and other high-grade gliomas. Ketogenic interventions varied, including the classic 4:1 ketogenic diet, modified Atkins diet, medium-chain triglyceride diets, and calorie-restricted or intermittent fasting protocols, often combined with standard treatments such as chemoradiotherapy or temozolomide. Duration of interventions ranged from days to over two years. Survival outcomes, including overall survival (OS) and progression-free survival (PFS), were reported variably across studies. Adverse events were mostly mild, with gastrointestinal symptoms, weight loss, and fatigue being the most common. No grade 3/4 toxicities directly related to the ketogenic diet were reported. Full details of study design, population, intervention type, outcomes, and adverse events are presented in (Supplementary Table 3).

### Narrative synthesis

Over the past three decades, there has been a steady increase in interest in using ketogenic dietary interventions (KD) to target altered tumor metabolism in gliomas. Pilot trials, observational cohorts, and more organized feasibility studies have progressively replaced the initial isolated case observations.

Nebeling et al. (1995) reported the first clinical evidence when they used an 8-week ketogenic diet high in medium-chain triglyceride oil in addition to standard therapy to treat two pediatric patients with advanced malignant astrocytomas. Positron emission tomography (PET) revealed a 21.8% decrease in tumor glucose uptake in both kids, along with an unexpectedly long survival of 60 and 48 months, respectively, and no notable side effects. This seminal study offered preliminary clinical evidence that metabolic modification could affect glioma tumor biology [12]. A remarkable case involving a 65-year-old woman with multicentric glioblastoma multiforme (GBM) who was treated with a strict 4:1 ketogenic diet along with therapeutic fasting in addition to conventional therapy was reported by Zuccoli et al. (2010) more than ten years later. Both MRI and PET imaging showed complete radiological remission. The study crucially showed that even strict ketogenic regimens could be used safely in GBM patients, with hyperuricemia being the only documented side effect [13]. Through a number of observational and pilot studies involving patients with high-grade gliomas, clinical experience with KD increased between 2014 and 2016. 11 patients treated with KD had a mean survival of 38 ± 13 months, according to Han et al. [14]. Concurrently, Rieger et al.'s ERGO study evaluated feasibility in 20 patients with recurrent GBM and confirmed that dietary ketosis could be attained, albeit with limited benefits for survival [15]. A median overall survival of 14 months was found in a larger retrospective analysis by Champ et al. that included 134 patients with grade III–IV gliomas [16]. According to a 2015 case report by Schwartz et al., two patients with WHO grade IV anaplastic astrocytoma were treated with an energy-restricted ketogenic diet [17]. Although survival and progression data were not disclosed, the diet was implemented over a 12-week period. There were no negative effects mentioned, and the report's main focus was on metabolic response and feasibility.

Patients with recurrent GBM treated with KD alone or in conjunction with bevacizumab showed disease stabilization, according to Artzi Moran et al. [18]. Santos et al. (Brazil) combined KD with intranasal perillyl alcohol in 37 recurrent GBM patients [19] and Martin-McGill et al. (UK, 2018) enrolled six glioma patients aged 16–69 on a restrictive KD for 3 months. The study focused on safety and adherence [20]. A 2018 prospective study by Van der Louw et al. evaluated the feasibility and clinical outcomes of a carbohydrate-restricted ketogenic diet in pediatric patients with diffuse intrinsic pontine glioma (DIPG0. The study included three patients, aged 4.4, 11.5, and 14.5 years, respectively. Individual overall survival times were 16.5, 6.4, and 18.7 months. Several side effects were observed, including hypoglycemia, hyperkeratosis, vomiting, refusal to eat, asthenia, and constipation. Despite these adverse effects, the study demonstrated that implementation of a carbohydrate-restricted ketogenic diet in pediatric DIPG patients is feasible [21]. In a 2018 case study, Elsakka and associates described how a 38-year-old patient with glioblastoma multiforme (GBM) used a calorie-restricted ketogenic diet. The long-term viability of metabolic therapy in the treatment of an aggressive brain tumor was investigated in this single-patient intervention. For an extended period of 24 months, the patient adhered to a rigorously calorie-restricted ketogenic diet while undergoing concurrent clinical monitoring. The report did not indicate whether chemotherapy or radiation was given concurrently with the diet, but it was implemented in conjunction with standard care measures. The patient continued to follow the dietary plan during the 24-month follow-up period [22].

Research began to concentrate more on integrating ketogenic techniques with conventional cancer treatments. The safety of KD during radiotherapy and adjuvant treatment phases was further supported by additional feasibility studies and case series by van der Louw et al., Woodhouse et al., and Berrington et al. [23–25]. Tóth et al. (2019) published a particularly noteworthy report that detailed a patient with recurrent GBM who was treated solely with a paleolithic ketogenic diet, without the use of chemotherapy or radiation. The patient's 46-month overall survival and 38 months of progression-free survival raise the intriguing possibility that, in carefully chosen cases, metabolic therapy alone may be beneficial [26]. In 2020, Martin-McGill et al. enrolled 12 patients with GBM (median age: 57) in a trial comparing MKD and MCTKD over 3 months. Median survival was 67.3 weeks. Electrolyte imbalances and constipation were the most common side effects [27].

Studies became more methodologically structured after 2020, frequently using intermittent fasting and calorie restriction. In one treatment arm, Klein et al.'s evaluation of a traditional 4:1 ketogenic diet as complete meal replacement in patients with terminal GBM revealed longer survival [28]. Panhans et al.'s retrospective analyses consistently confirmed an acceptable safety profile despite highlighting significant variability in outcomes [29]. Short-term calorie-restricted ketogenic diets combined with intermittent fasting were shown to be metabolically safe and feasible in patients with recurrent GBM in the ERGO2 studies by Voss et al. and Wenger et al. [30, 31]. Foppiani and colleagues (2020) ran a single-arm prospective study in Italy with six patients (median age: 45 years) diagnosed with high-grade gliomas. Over one month on an isocaloric KD, participants showed a 5% reduction in both body weight and waist circumference, suggesting effective metabolic engagement. Adverse effects and survival outcomes were not detailed [32]. Schreck et al. conducted a prospective study in 25 patients with astrocytomas (grade 2 to 4) who followed the Glioma Atkins-Based Diet for eight weeks. Although survival outcomes weren’t reported, the diet was associated with several adverse events. These included grade 2 leukopenia, nausea, diarrhea, fatigue, and seizures, along with one case of grade 3 neutropenia [33]. In a smaller retrospective study of five glioma patients, Perez et al. found a median survival of 18.7 months under ketogenic dietary therapy. Adverse events included hypoglycemia, constipation, vomiting, asthenia, hyperkeratosis, and hyperuricemia, highlighting the need for close monitoring even in small-scale implementations [34].

A patient with IDH-mutant GBM treated with ketogenic therapy alone, without radiation or chemotherapy, had an amazing 80-month overall survival, according to Seyfried et al. [35]. When KD was used in conjunction with standard treatment, phase-1study by Porper et al. showed sustained ketosis, good adherence, and manageable toxicity [36]. In a separate randomized trial, Voss et al. investigated the safety of a short, calorie-restricted ketogenic diet in 50 patients with recurrent glioblastoma [37]. Patients followed the diet for just three days prior to resuming standard therapy, with follow-up ranging from 250 to 485 days. While muscle cramps, gastrointestinal discomfort, and headaches were noted, these effects were temporary, suggesting that brief KD cycles can be tolerated even in advanced-stage disease. In a prospective case series from New Zealand, Phillips et al. enrolled ten patients with GBM who followed a modified ketogenic diet for an average of 127± 59 days [38]. The median survival was 13 months, in line with standard treatment expectations. The diet was well tolerated, with only mild side effects such as fatigue, irritability, and lightheadedness, and no grade 3 or higher toxicities were reported. A 2022 prospective study by Schwartz et al. (USA) investigated the feasibility of a ketogenic diet in patients with GBM. The study enrolled 12 patients. Participants followed the ketogenic diet for six weeks, with no clearly defined follow-up duration reported [39].

Smith K and colleagues conducted a retrospective observational study in the U.S., evaluating ketogenic metabolic therapy in 16 glioma patients. The cohort included a mix of WHO grade II–IV gliomas. KMT was provided as a standalone intervention over an average of 20.6 months. Mean progression-free survival was 20.0 months. The intervention was safe, with no diet-related adverse events reported [40]. A 64-year-old patient with IDH-wildtype glioblastoma was treated with a time-restricted ketogenic diet, according to a more recent 2024 case report by Phillips et al. With an overall survival of 36 months, the diet was maintained for two years, which is much higher than the median for this molecular subtype. During extended fasting, mild side effects such as fatigue, diarrhea, and cold intolerance were observed; however, no adverse events directly linked to KD were reported [41]. A multicenter observational study in adults with high-grade gliomas was recently published by Laguado et al. (2024), which confirmed the long-term viability, good tolerability, and lack of serious adverse events during a 12-month ketogenic intervention [42]. A 2025 prospective study by Kiryttopoulos et al. (USA) evaluated the use of a ketogenic diet in patients with newly diagnosed GBM. The study included 18 patients with a median age of 57.5 years, who followed a ketogenic diet for six months, with a reported follow-up period of six months. Among the sub-group with available long-term outcome data, the 3-year survival rate was 66.7%, which appears higher than typically reported for glioblastoma [8]. At Cedars-Sinai Medical Center, Amaral and colleagues carried out an open-label study involving 20 glioblastoma patients. The ketogenic intervention was administered alongside temozolomide chemotherapy for 16 weeks. Outcomes were promising, with a median overall survival of 29.4 months and a progression-free survival of 12.9 months. Importantly, the diet was well tolerated, with no reported adverse events or discontinuations [7].

In abstracts, we found that William M and colleagues (UK) initiated two parallel, open-label Phase 2 trials to explore modified KD (MKD) in both high- and low-grade glioma patients. The MKD was planned in conjunction with standard chemoradiotherapy for high-grade gliomas or standard care for low-grade gliomas. Data from these trials are pending [43]. Shen et al. (2016), who successfully used a modified ketogenic formula during chemo-radiation in a child with GBM, provided additional evidence of feasibility in pediatric settings [44]. Across these studies, KD was generally well tolerated, with adverse effects largely limited to weight loss, gastrointestinal symptoms, and occasional hypoglycemia. With an overall survival of 29.3 months, Dardis et al. (2017) showed that starting KD during chemo-radiation and subsequently switching to a Modified Atkins Diet was both practical and well tolerated [45]. Gresham G and team (2019) began an open-label feasibility trial in 20 newly diagnosed GBM patients. The 16-week KD was introduced during standard therapy. Full outcome data were not yet available, but preliminary results indicated feasibility and tolerability [46]. Schwartz K et al. (2019) conducted a pilot trial in nine GBM patients on a 3:1 isocaloric KD during standard therapy. Younger patients had no tumor progression at 31–49 months, while older patients experienced shorter survival. The KD was safe and well tolerated [47]. N. Shinojima et al. described the clinical course of a 37-year-old male patient with progressive GBM who was treated with palliative care in addition to a ketogenic diet in a case report from Japan. After standard therapies showed signs of disease progression, dietary intervention was started. Although the composition of the KD regimen was not specified, it was followed for a minimum of eight months, and an MRI at that time revealed a favorable therapeutic response. The patient remained clinically stable under the ketogenic protocol for the entire 20-month clinical follow-up period following the diagnosis. Crucially, during the course of treatment, the patient did not encounter any negative consequences related to the ketogenic diet [48]. Smiti S (Morocco) ran a small prospective study comparing KD during radiotherapy versus usual diet in 10 GBM patients over three months [49]. Nelson T et al. (2021) shared early findings from a U.S. Phase 1 trial involving 14 newly diagnosed GBM patients (median age: 55). Participants followed KD for 16 weeks alongside standard therapy. Target ketone levels (> 0.3 mmol/L) were maintained. The median overall survival at the time of reporting was 14.6 months, consistent with standard care [50].

The cumulative clinical evidence points to the viability, general safety, and biological activity of ketogenic dietary interventions for glioma patients. Firm conclusions regarding efficacy are limited by significant heterogeneity in dietary protocols, study designs, and outcome reporting, even though sporadic reports describe remarkably prolonged survival, particularly in selected patients with favorable molecular profiles. These results highlight the critical need for carefully planned, sufficiently powered randomized controlled trials to validate the actual therapeutic role of ketogenic approaches in the treatment of gliomas.

### Joanna Briggs Institute (JBI) assessment

All included studies were rated as high methodological quality using JBI appraisal tools, with scores ranging from 77 to 100%, and were of high quality. Minor limitations included lack of blinding in non-randomized studies and unclear consecutive inclusion in some case series. No studies were excluded based on quality. The complete detail is given in (Supplementary Table 4).

## Discussion

This systematic review compiles the clinical evidence published between 1995 and 2025, reassessing the efficacy and safety of the ketogenic diet as an adjunctive metabolic therapy for glioblastoma multiforme. The findings extend the conclusions which showed strong feasibility and metabolic rationale for ketogenic therapy, with limited data on survival benefit [6]. With the inclusion of earlier and recent prospective trials, the present synthesis exposes consistent patterns that nutritional ketosis may contribute to prolonged progression-free survival, improved overall survival, and enhanced tolerability when combined with GBM standard treatment regimens[7, 8]. GBM is still the most common malignant primary brain tumor, accounting for over 50% of all malignant central nervous system neoplasms, with an age-adjusted U.S. incidence of 3.27/100,000 and median survival of only 14.6 months under the Stupp protocol [1–4]. Even after maximal safe resection followed by radiotherapy and temozolomide, outcomes remain poor due to the tumor’s marked heterogeneity and metabolic adaptability. A hallmark characteristic of GBM is its abnormal metabolic phenotype represented by the Warburg effect which is preferential utilization of aerobic glycolysis despite adequate oxygenation, which confers a “glucose addiction” driving aggressive proliferation and therapeutic resistance [5, 9]. This dependency offers a unique metabolic vulnerability which can be targeted by the ketogenic diet via the induction of a fasting-like state that reduces systemic glucose and insulin levels while increasing ketone bodies such as β-hydroxybutyrate, selectively starving the metabolically inflexible tumor cells [9, 15]. Over the 38 studies analyzed, adherence to KD was generally high, with over 75% of patients achieving and maintaining nutritional ketosis (β-hydroxybutyrate ≥ 0.5 mmol/L). This is in line with the feasibility data from Amaral et al. in 2025 [7] who reported 79% compliance during concurrent chemoradiotherapy and reported median PFS and OS of 12.9 and 29.4 months, respectively-nearly twice the survival expected under standard-of-care protocols[3, 4]. Similarly, Kiryttopoulos et al., in 2025 [8], presented 66.7% 3-year survival in adherent patients undergoing ketogenic metabolic therapy versus 8.3% in controls, with minimal grade ≥ 2 adverse effects. Early pioneering reports such as Nebeling et al. [12] and Zuccoli et al. [13] had already suggested that ketogenic metabolic therapy could induce radiographic tumor regression and significantly extend survival beyond conventional expectations, findings now corroborated by multiple modern studies. Together, these data substantiate the hypothesis that KD may synergize with existing oncologic modalities to suppress GBM progression. A conspicuous feature of this review is the increasingly eclectic mix of ketogenic interventions employed in studies. While the classical 4:1 fat-to-carbohydrate-plus-protein ratio diet remains the gold standard, newer regimens such as the modified Atkins diet, calorie-restricted ketogenic diet, and intermittent fasting variants exhibited equal metabolic efficacy [30, 38]. A randomized trial ERGO2 by Voss et al. [38] established a calorie-restricted KD in conjunction with reirradiation in recurrent glioma patients to be safe and associated with improved metabolic profiles and survival trends compared to controls. Equally, Martin-McGill et al. [34] showed the deliverability and tolerability ofKD within a national health service. These findings taken together suggest that KD can be adapted to various clinical and logistic needs, expanding its putative indications throughout disease courses and patient populations. In terms of overall survival, all the studies combining KD with the Stupp regimen consistently reported extended PFS and OS compared to historical benchmarks. Amaral et al. [7] achieved median OS of 29.4 months versus 14.6 months under standard therapy, while Rieger et al. [15] and Phillips et al. [38] observed disease stabilization and longer survival in recurrent GBM cohorts. Dal Bello et al. [18] similarly concluded that metabolic therapy may prolong OS when combined with conventional treatment, particularly when adherence and sustained ketosis are achieved. Noticeably, studies utilizing concurrent metabolic and radiotherapeutic interventions (Voss et al.) [30] or low-glucose index monitoring (Meidenbauer et al.) [51] demonstrated a dose-dependent relationship between the intensity of ketosis and survival benefit, supporting the concept that the therapeutic effect of KD may be dependent upon metabolic engagement. Adverse effects of KD across studies were uniformly mild, while gastrointestinal disturbances, weight loss, and fatigue were the most common. No severe or grade ≥ 3 toxicities directly related to the diet were recorded, further supporting its safety profile [6–8]. Martin-McGill et al. [52] and Phillips et al. [38] independently reported maintained or improved quality of life with KD, including increased energy and mental clarity. Pediatric and palliative cases also proved feasible in advanced or refractory disease, suggesting that KD may have utility beyond first-line therapy [Perez et al., [34]; Seyfried et al., [35]]. Taken together, these data confirm that ketogenic therapy represents a safe and well-tolerated adjunctive approach in GBM management. At a mechanistic level, the therapeutic rationale underlying KD is increasingly well-characterized. By limiting glucose availability and inducing ketosis, KD impairs glycolytic flux through inhibition of Pyruvate Kinase Isoenzyme M2, downregulates Hypoxia-Inducible Factor 1-alpha, and activates Adenosine Monophosphate-Activated Protein Kinase pathways, leading to decreased proliferation and increased apoptosis in tumor cells [5, 9, 53]. KD also impacts epigenetic regulation; ketone bodies modify histone deacetylases and influence the expression of metabolic and proapoptotic genes and more recently, Talib et al. [54] highlighted how KD may modulate oxidative stress and inflammation, central elements driving GBM pathophysiology. Furthermore, the GKI proposed by Meidenbauer et al. [51] provides a quantitative biomarker of metabolic control that may predict therapeutic response. Recent frameworks by Duraj et al. [9] emphasize integrating GKI monitoring into clinical trial design to optimize patient selection and diet intensity. These mechanistic insights collectively reinforce the hypothesis that KD exploits the unique metabolic vulnerabilities of GBM, enhancing therapeutic efficacy while sparing normal neuronal metabolism. Despite this, several limitations are still associated with the current evidence base. The majority of available studies are small, non-randomized, and heterogeneous in design, with variable macronutrient ratios, adherence assessment methods, and endpoints. Few trials included molecular profiling of Isocitrate Dehydrogenase mutation or O6-Methylguanine-DNA Methyltransferase methylation status, which may influence metabolic sensitivity and therapeutic outcomes [7, 8]. Although preliminary data suggest that IDH-mutant and MGMT-methylated tumors may prove more sensitive owing to diminished glycolytic flexibility, this needs validation in adequately powered phase III randomized trials. Moreover, issues of long-term sustainability, patient adherence, and metabolic adaptation require In conclusion, this comprehensive synthesis of clinical data spanning three decades provides compelling evidence that ketogenic metabolic therapy is feasible, safe, and biologically rational adjunct to current glioblastoma management. Consistent improvement trends in survival, metabolic control, and quality of life, combined with negligible toxicity, underpin KD's therapeutic promise. Building upon the frameworks proposed by Duraj et al. [9] and further supported by the metabolic insights of Irin et al. [5], ketogenic therapy represents a viable strategy in targeting GBM's hallmark glycolytic dependency. Future well-powered, multicenter randomized trials incorporating standardized metabolic biomarkers, molecular stratification, and uniform dietary protocols are essential in order to validate KD's role and establish evidence-based clinical guidelines.

A critical consideration when interpreting the survival outcomes across studies is the substantial heterogeneity among the included investigations. Dietary interventions differed considerably, ranging from classical ketogenic diets with a four to one ratio of fat to combined carbohydrate and protein to modified Atkins diets, calorie restricted ketogenic diets, medium chain triglyceride based diets, and intermittent fasting protocols. These variations result in differences in the degree and duration of ketosis achieved, caloric intake, and patient adherence, which may influence metabolic engagement and treatment response. In addition, the studies varied in terms of concurrent therapies administered alongside ketogenic interventions, including radiotherapy, temozolomide chemotherapy, antiangiogenic agents, and in some cases ketogenic therapy alone. Patient populations were also heterogeneous, including newly diagnosed glioblastoma multiforme, recurrent tumors, pediatric high grade gliomas, and mixed cohorts of glioma grades. Differences in disease stage, molecular tumor characteristics, and prior treatment exposure further complicate the interpretation of pooled survival data. Consequently, although several studies report prolonged survival with ketogenic metabolic therapy, the heterogeneity in diet protocols, treatment combinations, and patient selection limits the ability to draw definitive conclusions regarding the magnitude of therapeutic benefit.

### Clinical implication

Cumulatively, it is evident that the ketogenic diet may potentially have synergistic effects on current treatment strategies and utilize the glycolysis dependency and metabolically rigid nature of glioblastoma multiforme cancer cells for better treatment outcomes. The addition of ketogenic metabolic therapies to conventional treatment strategies may potentially improve the sensitivity to radiation and minimize systemic toxicities associated with insulin and glucose-driven growth signaling. Additionally, the feasibility and applicability associated with ketogenic therapies may provide a cost-effective and non-toxic treatment option for treatment-resistant cancer patients. Potentially applicable to patients at all ages, including both younger and older patients, it can be utilized to potentially monitor metabolic control parameters such as glucose and ketone ratio.

### Future directions

Future studies ought to focus on large-scale multicenter randomized clinical trials incorporating standardized dietary approaches, metabolic profiling, and molecularly defined stratification. Molecularly defined stratification, according to patients' Isocitrate Dehydrogenase mutation and O6-Methylguanine-DNA Methyltransferase methylation status, would allow for identification of genetic predictors for metabolism-driven response [7, 8]. In addition, investigating combination therapies that combine a ketogenic diet with immunotherapy, antiangiogenic therapies, and inhibitors of glutamine metabolism may potentially maximize treatment responses. Data regarding neurocognitive and metabolic compliance and treatment response longitudinal data would also be crucial towards long-term feasibility. The framework established by a study by Duraj et al. (2024), provides a sys.

#### Ongoing and registered clinical trials

An important forward-looking aspect of the current evidence base is the increasing number of ongoing or registered randomized clinical trials investigating ketogenic metabolic therapy in patients with gliomas. Several prospective studies are currently evaluating modified ketogenic dietary protocols in combination with standard chemoradiotherapy for both high grade and low grade gliomas. These trials aim to determine whether sustained nutritional ketosis can improve progression free survival, overall survival, and patient reported quality of life outcomes compared with standard therapy alone. The availability of randomized controlled trial data will be crucial in clarifying the true clinical efficacy of ketogenic metabolic therapy and in establishing standardized dietary protocols for future clinical practice.

Building on these promising results, several larger and more rigorous trials have now been initiated. A search of clinical trial registries reveals several studies designed to address the evidence gaps highlighted in the current literature.

Ketogenic Diet and Metformin (NCT04691960): This pilot study at Weill Medical College of Cornell University is investigating a combination approach. It assesses the feasibility and tolerability of adding metformin to a continuous ketogenic diet in patients with high-grade gliomas. The study provides crucial data on the safety and metabolic effects of combining two glucose-targeting strategies [55].

DIET2TREAT (NCT05708352): This is a randomized, multicenter Phase 2 trial aiming to enroll 170 patients with newly diagnosed GBM. It randomly assigns participants 1:1 to either a strict ketogenic diet or standard dietary guidance for 18 weeks, alongside standard chemoradiation. The primary outcome is overall survival, with extensive secondary outcomes including progression-free survival, quality of life, cognitive performance, and metabolic biomarkers. The trial is active at multiple U.S. sites, including Cedars-Sinai and UCSF [56].

Metabolic Therapy Program (NCT04730869): Being conducted at Waikato Hospital in New Zealand, this trial is evaluating a more intensive metabolic therapy program. It combines standard chemoradiation with repeated 5-day fasts and a time-restricted, modified ketogenic diet. The study focuses on feasibility, safety, and its effect on the glucose-to-ketone ratio, with survival as a secondary outcome. Its preliminary completion date was February 2025, with results expected soon [57].

The results of these and other registered trials will be critical in determining whether the survival benefits observed in smaller cohorts can be replicated in larger, controlled populations and will help establish standardized protocols for clinical implementation. A thematic format for aligning treatment strategies and demarcating a specific clinical endpoint for metabolic therapies.

## Limitations

The present body of evidence is limited by small cohort numbers, lack of blinding, and variability in diet composition, treatment length, and levels of diet compliance. Most trials were conducted in an observational fashion, making it difficult to establish causality and rendering these trials vulnerable to selection bias. Moreover, the long-term feasibility of following very low carbohydrate diets is likely to remain a challenge for patients. The availability of only limited registered dietitians competent in ketogenic treatment and varying levels of patient compliance have been other challenges. Additionally, there is no common set of biomarkers for the following treatment response.

## Conclusions

This integrative analysis of clinical and mechanistic findings strongly supports the role of the ketogenic diet as a practicable, safe, and mechanistically plausible adjunctive therapy for glioblastoma multiforme. The approach exploits deep biochemical vulnerabilities such as undue glucose overexpression and oxidative phosphorylation dysfunction, thus offering a complementary approach to existing chemoradiation regimens. The recent failure to observe any meaningful toxicities, and a trend toward enhanced survival and quality of life, collectively indicate that its formal assessment within controlled clinical trials is warranted. Going forward, individualized metabolic therapies defined according to precise molecular and metabolic characterization could completely redefine the treatment of glioblastoma multiform and regularize the ketogenic diet as a proven metabolic therapy.

## Supplementary Information

Below is the link to the electronic supplementary material.

Supplementary file1 (DOCX 86 KB)

## Abbreviations

CNS: Central nervous system
BHB: β-Hydroxybutyrate
Akt/mTOR: Protein kinase B/mammalian target of rapamycin
DIPG: Diffuse intrinsic pontine glioma
GBM: Glioblastoma / glioblastoma multiforme
GKI: Glucose ketone index
GLUT1: Glucose transporter 1
HGG: High-grade glioma
HIF-1α: Hypoxia-inducible factor-1 alpha
IDH: Isocitrate dehydrogenase
JBI: Joanna Briggs Institute
KD: Ketogenic diet
KMT: Ketogenic metabolic therapy
LGG: Low-grade glioma
MCT: Medium-chain triglyceride
MCTKD: Medium chain triglyceride ketogenic diet
MGMT: O6-methylguanine-DNA methyltransferase
MKD: Modified ketogenic diet
MRI: Magnetic resonance imaging
OS: Overall survival
PET: Positron emission tomography
PFS: Progression-free survival
PKM2: Pyruvate kinase muscle isozyme 2
POH: Perillyl alcohol
PRISMA: Preferred reporting items for systematic reviews and meta-analyses
RT: Radiotherapy
TMZ: Temozolomide
TTP: Time to progression
WHO: World health organization
