# Supplementary material for: Efficacy and safety of ketogenic diet in glioblastoma: an updated systematic review and meta-analysis
Source: Neurol Sci. 2026 Apr 25;47(5):461. doi: 10.1007/s10072-026-09035-y (PMC13109122; doi:10.1007/s10072-026-09035-y)
Supplement: Supplementary file 1 — Supplementary file1 (DOCX 86 KB) [file 10072_2026_9035_MOESM1_ESM.docx]

**Efficacy and Safety of Ketogenic Diet in Glioblastoma: An updated systematic review**

**Supplementary Table 1.** PRISMA checklist for the systematic review and meta-analysis.

**Supplementary Table 2.** Search strategy and databases used for literature selection.

**Supplementary Table 3:** Baseline Characteristics of Included Studies

**Supplementary Table 4:** Joanna Briggs Institute (JBI) Critical Appraisal Checklist

**Supplementary Table 1.** PRISMA checklist for the systematic review and meta-analysis.

| **Section and Topic** | **Item #** | **Checklist item** | **Location where item is reported** |
| --- | --- | --- | --- |
| **TITLE** | | |  |
| Title | 1 | Identify the report as a systematic review. | Page 1 |
| **ABSTRACT** | | |  |
| Abstract | 2 | See the PRISMA 2020 for Abstracts checklist. | Page 2 |
| **INTRODUCTION** | | |  |
| Rationale | 3 | Describe the rationale for the review in the context of existing knowledge. | Page 3 |
| Objectives | 4 | Provide an explicit statement of the objective(s) or question(s) the review addresses. | Page 3 |
| **METHODS** | | |  |
| Eligibility criteria | 5 | Specify the inclusion and exclusion criteria for the review and how studies were grouped for the syntheses. | Page 4 |
| Information sources | 6 | Specify all databases, registers, websites, organisations, reference lists, and other sources searched or consulted to identify studies. Specify the date when each source was last searched or consulted. | Page 4 |
| Search strategy | 7 | Present the full search strategies for all databases, registers, and websites, including any filters and limits used. | Page 4 , Supplementary Table 2 |
| Selection process | 8 | Specify the methods used to decide whether a study met the inclusion criteria of the review, including how many reviewers screened each record and each report retrieved, whether they worked independently, and, if applicable, details of automation tools used in the process. | Page 4 |
| Data collection process | 9 | Specify the methods used to collect data from reports, including how many reviewers collected data from each report, whether they worked independently, any processes for obtaining or confirming data from study investigators, and, if applicable, details of automation tools used in the process. | Page 4 |
| Data items | 10a | List and define all outcomes for which data were sought. Specify whether all results that were compatible with each outcome domain in each study were sought (e.g., for all measures, time points, analyses), and if not, the methods used to decide which results to collect. | Page 4 |
|  | 10b | List and define all other variables for which data were sought (e.g., participant and intervention characteristics, funding sources). Describe any assumptions made about any missing or unclear information. | Page 4 |
| Study risk of bias assessment | 11 | Specify the methods used to assess risk of bias in the included studies, including details of the tool(s) used, how many reviewers assessed each study, and whether they worked independently, and if applicable, details of automation tools used in the process. | Page 9 |
| Effect measures | 12 | Specify for each outcome the effect measure(s) (e.g., risk ratio, mean difference) used in the synthesis or presentation of results. | Page 4,6 |
| Synthesis methods | 13a | Describe the processes used to decide which studies were eligible for each synthesis (e.g., tabulating the study intervention characteristics and comparing against the planned groups for each synthesis (item #5)). | Page 4 |
|  | 13b | Describe any methods required to prepare the data for presentation or synthesis, such as handling of missing summary statistics or data conversions. | Page 5 |
|  | 13c | Describe any methods used to tabulate or visually display the results of individual studies and syntheses. | Page5 |
|  | 13d | Describe any methods used to synthesize results and provide a rationale for the choice(s). If meta-analysis was performed, describe the model(s), method(s) to identify the presence and extent of statistical heterogeneity, and software package(s) used. | Page 5 |
|  | 13e | Describe any methods used to explore possible causes of heterogeneity among study results (e.g., subgroup analysis, meta-regression). | Page 10 |
|  | 13f | Describe any sensitivity analyses conducted to assess the robustness of the synthesized results. | N/A |
| Reporting bias assessment | 14 | Describe any methods used to assess the risk of bias due to missing results in a synthesis (arising from reporting biases). | Page 11 |
| Certainty assessment | 15 | Describe any methods used to assess certainty (or confidence) in the body of evidence for an outcome. | N/A |
| **RESULTS** | | |  |
| Study selection | 16a | Describe the results of the search and selection process, from the number of records identified in the search to the number of studies included in the review, ideally using a flow diagram. | Page 5 |
|  | 16b | Cite studies that might appear to meet the inclusion criteria, but which were excluded, and explain why they were excluded. | Page 5 |
| Study characteristics | 17 | Cite each included study and present its characteristics. | Page 6 |
| Risk of bias in studies | 18 | Present assessments of risk of bias for each included study. | Page 9 |
| Results of individual studies | 19 | For all outcomes, present, for each study: (a) summary statistics for each group (where appropriate) and (b) an effect estimate and its precision (e.g., confidence/credible interval), ideally using structured tables or plots. | Page 6-9 |
| Results of syntheses | 20a | For each synthesis, briefly summarise the characteristics and risk of bias among contributIn studies. | Page 6,9 |
|  | 20b | Present the results of all statistical syntheses conducted. If meta-analysis was done, present for each the summary estimate and its precision (e.g., confidence/credible interval) and measures of statistical heterogeneity. If comparing groups, describe the direction of the effect. | Page 6-9 |
|  | 20c | Present the results of all investigations of possible causes of heterogeneity among study results. | Page 10 |
|  | 20d | Present the results of all sensitivity analyses conducted to assess the robustness of the synthesized results. | Page N/A |
| Reporting biases | 21 | Present assessments of risk of bias due to missing results (arising from reporting biases) for each synthesis assessed. | Page 11 |
| Certainty of evidence | 22 | Present assessments of certainty (or confidence) in the body of evidence for each outcome assessed. | Page 12 |
| **DISCUSSION** | | |  |
| Discussion | 23a | Provide a general interpretation of the results in the context of other evidence. | Page 10 |
|  | 23b | Discuss any limitations of the evidence included in the review. | Page 11 |
|  | 23c | Discuss any limitations of the review processes used. | Page 11 |
|  | 23d | Discuss implications of the results for practice, policy, and future research. | Page 11 |
| **OTHER INFORMATION** | | |  |
| Registration and protocol | 24a | Provide registration information for the review, including register name and registration number, or state that the review was not registered. | Page 4 |
|  | 24b | Indicate where the review protocol can be accessed, or state that a protocol was not prepared. | Page 4 |
|  | 24c | Describe and explain any amendments to information provided at registration or in the protocol. | N/A |
| Support | 25 | Describe sources of financial or non-financial support for the review, and the role of the funders or sponsors in the review. | Page 13 |
| Competing interests | 26 | Declare any competing interests of review authors. | Page 13 |
| Availability of data, code, and other materials | 27 | Report which of the following are publicly available and where they can be found: template data collection forms; data extracted from included studies; data used for all analyses; analytic code; any other materials used in the review. | Page 13 |

**Supplementary Table 2.** Search strategy and databases used for literature selection.

| **Database / Source** | **Search String** | **Total number(n)** |
| --- | --- | --- |
| **PubMed** | ("Glioblastoma"[Mesh] OR "Glioma"[Mesh] OR "Astrocytoma"[Mesh] OR glioblastoma OR "glioblastoma multiforme" OR "high grade glioma" OR "grade III glioma" OR "grade IV glioma" OR GBM OR HGG OR "astrocytoma" OR "anaplastic astrocytoma" OR "gliosarcoma" OR "malignant brain tumor" OR "malignant brain neoplasm" OR "brain glioma" OR "brain tumor" OR "brain neoplasm") ) AND (ketogenic OR "ketogenic diet" OR "ketone diet" OR ketosis OR "ketone body" OR "ketone therapy" OR "ketogenic therapy" OR "modified Atkins" OR "Atkins diet" OR "medium chain triglyceride" OR "MCT diet" OR "low carbohydrate" OR "low carb" OR "low carbohydrate high fat" OR "low carb high fat" OR "high fat diet" OR "fat-based diet" OR "metabolic therapy" OR "calorie restriction" OR "restricted calorie diet" OR "energy restriction" OR "nutritional intervention" OR "dietary intervention") | 206 |
| **Cochrane Library (CENTRAL)** | ( Glioblastoma OR Glioma OR Astrocytoma OR "glioblastoma multiforme" OR ("high" NEAR/2 "grade" NEAR/2 glioma) OR ("grade" NEXT "III" NEAR/2 glioma) OR ("grade" NEXT "IV" NEAR/2 glioma) OR GBM OR HGG OR ("malignant" NEAR/3 ("brain" NEAR/3 (tumor OR neoplasm OR cancer))) OR astrocytoma OR "anaplastic astrocytoma" ) AND ( "Ketogenic Diet" OR "Diet, Fat-Restricted" OR ("ketogenic" NEAR/3 (diet OR therapy OR intervention OR regimen OR nutrition)) OR ("low" NEAR/3 ("carbohydrate" NEAR/3 ("high" NEAR/2 "fat"))) OR ("low-carbohydrate" NEAR/3 ("high-fat" OR "high fat")) OR ("modified" NEAR/2 "Atkins") OR ("medium chain triglyceride" NEAR/2 (diet OR therapy)) OR ("calorie" NEAR/2 ("restriction" OR "restricted")) OR ("energy" NEAR/2 ("restriction" OR "restricted")) OR "metabolic therapy" OR ketosis OR "ketone body" ) | 37 |
| **Web of Science** | TS = (("glioblastoma" OR "glioblastoma multiforme" OR "high grade glioma" OR GBM OR HGG) AND ("ketogenic diet" OR "ketone diet" OR "modified Atkins" OR "medium chain triglyceride diet" OR "low carbohydrate high fat" OR "low-carbohydrate diet" OR ketosis OR ketogenic) | 172 |
| **Embase** | ('glioblastoma'/exp OR 'high grade glioma'/exp OR glioblastoma OR 'glioblastoma multiforme' OR gbm  OR hgg) AND ('ketogenic diet'/exp OR 'modified  atkins diet'/exp OR 'low carbohydrate diet'/exp  OR ketogenic OR 'ketogenic therapy' OR ketosis OR  'low carbohydrate high fat' OR 'mct diet' OR  'medium chain triglyceride diet') | 424 |

**Supplementary Table 3:** Baseline Characteristics of Included Studies

| **Study ID** | **Location** | **Study design** | **Sample size** | **Age**  **(years)** | **Diagnosis(Grade and disease stage)** | **Interventions** | **KD duration(months,range)** | **Follow up Time(months,[range])** | **Survival Data(months,[range])** | | **Adverse events related to KD** |
| --- | --- | --- | --- | --- | --- | --- | --- | --- | --- | --- | --- |
| **Nebeling et al., 1995**  (12) | N/A | Case Report | 2 | N/A | Advanced-stage malignant Astrocytoma tumors | 60% medium chain triglyceride oil-based diet | 8 weeks | 8 weeks in both patients; up to 12 months of extended follow-up in one patient | 60 months and 48 months | N/A | N/A |
| **Zuccoli et al., 2010**  **(13)** | N/A | Case Report | 1 | 65 year | Multicentric Glioblastoma Multiforme | Restricted ketogenic diet | 2 months | 2 months | NA | N/A | Hyperurecemia |
| **Han et al., 2014**  **(14)** | N/A | Prospective Study | 11 | N/A | N/A | N/A | N/A | N/A | Mean Survival: 38 +/− 13 months | N/A | N/A |
| **Rieger et al., 2014**  (15) | Germany | Prospective Study | 20 | 57 (30-72) | recurrent  glioblastoma | ketogenic diet | 6-8-week | 6-8-week | Median: 32 weeks | N/A | Weight loss, diarrhea, constipation, hunger |
| **Champ et al., 2014**  (16) | NY | Retrospective Analysis | 134 | N/A | grade III–IV gliomas | Ketogenic diet | N/A | 14 months | Median: 14 months | N/A | Constipation, asthenia, weight loss, nephrolithiasis,  hypoglycemia |
| **Schwartz et al., 2015**  (17) | N/A | Case Report | 2 | Patient no. 1, a 55-year-old Patient no. 2, a 52-year-old | IV anaplastic astrocytoma | energy-restricted ketogenic diet | N/A | 12 weeks | NA | N/A | N/A |
| **Artzi Moran 2016** | Israel | Prospective & Retrospective Cohort | 9 | 51; 46 | 4 Recurrent GB, 1 Gliomatosis Cerebri, 4 Recurrent GB | 4 KD + Bevacizumab, 1 KD only, Control: Various (TMZ, Bevacizumab, Rindopepimut) | 2 to >31 (Variable) | 31 | Not explicitly reported for all. One patient (Gliomatosis) had stable disease for >31 months. | N/A | Ongoing weight loss was noted as a potential adverse effect. No major side effects reported. |
| **Santos et al., 2017**  (19) | Brazil | Prospective Randomized | 37 | median, 53 years old | recurrent  GBM | KD  with intranasal delivery of POH | N/A | N/A | NA | N/A | N/A |
| **Van der Louw et al.,** 2018  (21) | N/A | Prospective Study | 3 | pt 1 4.4 years pt2 11.5 pt3 14.5 | diffuse intrinsic pontine glioma (DIPG) | carbohydrate-restricted ketogenic diet (KD | N/A | 3 months | 16.5, 6.4, and 18.7 months | N/A | Hypoglycemia, hyperkeratosis, vomiting, refusal  to eat, asthenia, constipation |
| Martin-McGill et al., **2018**  (52) | UK | Prospective Study | 6 | aged between 16 and 69 years | Glioma | restrictive KD | 3 months | N/A | NA | N/A | Constipation |
| **Elsakka 2018**  (22) |  | Case report | 1 | 38-year-old | GBM | calorie  restricted ketogenic diet | 24 month | 24 month | N/A | N/A | N/A |
| **Van der Louw et al., 2019**  **(21)** | Netherland | Prospective Study | 11 | years, median 53.8 (22.5) | glioblastoma multiforme | full liquid KD | 6 week | 14 weeks | 9.8 and 19.0 months | N/A | Constipation, nausea/vomiting,  hypercholesterolemia, hypoglycemia, diarrhea,  low carnitine concentration |
| **Woodhouse et al., 2019**  (24) | USA | Retrospective Study | 29 | 30.8 years to 76.7 years | grades II–IV astrocytoma | modified Atkins diet | 6 week | 6 week | Not evaluated | N/A | Grade 2 constipation occurred in 1 patient. Grade 1  fatigue and nausea, probably due to the standard  therapy |
| **Berrington A 2019**  **(25)** | USA | single-arm Phase II | 10 | (mean) (SD) (yr) 49.2 (10.6) | Seven (70%)   had WHO grade III anaplastic astrocytomas, and 3 (30%) had   glioblastomas | Atkins-based ketogenic diet | 8 week | 8 week | N/A | N/A | N/A |
| **Tóth C 2019**  (26) | Budapest, Hungary | Case Report | 1 | Not Specified | Recurrent GB | PKD as a stand-alone therapy | 38 months | 38 months | 46 months | 38 months | No Adverse events |
| **Martin-McGill et al., 2020**  (27) | UK | Prospective Study | 12 | median age of 57 years (44–66 years) | GBM (WHO grade IV | modified ketogenic diet (MKD) or medium chain triglyceride ketogenic diet (MCTKD | 3 month | 3 month | Median: 67.3 weeks | N/A | Hypokalemia, hypocalcemia, hypernatremia,  hyperkalemia, constipation |
| **Klein et al., 2020**  (28) | USA | Prospective Randomized | 8 | mean age 49.8 years (range 40–64) | Terminal GBM | Ketogenic diet | 6 months | N/A | Group 1: 20 months (9.5–27)  Group 2: 12.8 months (6.3–19.9) | N/A | Weight loss, hunger, nausea, dizziness, asthenia,  constipation |
| **Panhans et al., 2020**  (29) | N/A | Retrospective Case Series | 12 | N/A | N/A | N/A | N/A | N/A | 90.8–19.0 Months | N/A | Asthenia, weight loss, nausea, vomiting, headache,  decreased appetite |
| **Voss et al., 2020**  (30) | Germany | Prospective Randomized | 50 | Median (Range) 56 (39-71) | gliosarcoma or malignant progression of a lower-grade glioma | calorically unrestricted diet or KD-IF | N/A | N/A | KD:331days  SD:291days  Low glucose KD: 348 days | N/A | Epileptic seizures, headache, nausea |
| **J. Wenger 2020**  (31) | Germany | RT | 19 | 51 | GBM | calorically restricted ketogenic diet/intermittent fasting (crKD-IF) | 9 days | 6 Days | N/A | N/A | N/A |
| **FoppianI A 2020**  (32) | Itlay | single-arm pro-  Prospective study | 6 | 45 (42; 46) | HGG | KD diet | 1 month | 1 week | N/A | N/A | Both body weight and waist circumference-  cumference were 5% lower after 1 mo of   intervention. |
| **Schreck et al., 2021**  (33) | UK | Prospective Study | 25 | (mean, SD), y 50.1 (12.7) | Grade 2 to 4 astrocytoma | Glioma  Atkins-Based Diet | 8 week | 8 week | N/A | N/A | Grade 2 adverse events: Leukopenia, nausea,  diarrhea, fatigue, or seizure. Grade 3: neutropenia  (possibly related) |
| **Perez et al., 2021**  (34) | N/A | Retrospective Study | 5 | N/A | N/A | N/A | N/A | N/A | Median: 18.7 Months | N/A | Hypoglycemia, constipation, hyperkeratosis,  vomiting, asthenia, hyperuricemia |
| **Seyfried et al., 2021**  (35) | UK | Case Report | 1 | 26-year- | IDH mutated Glioblastoma | low-carbohydrate ketogenic diet | 2 weeks | N/A | 80 Months | N/A | N/A |
| **Porper et al., 2021**  (36) | Israel | Prospective Randomized  Study | 13 | median age 61 years | diagnosed and recurrent gliomas | Modified Atkins Diet (ModAD) supplemented with medium-chain triglycerides (MCT) | N/A | N/A | 21 Months in patients with newly  diagnosed disease  8 Months in patients with  recurrent disease | N/A | Nausea, asymptomatic hyperuricemia, anorexia |
| **Voss et al., 2022**  (37) | Germany | Prospective Randomized | 50 | N/A | recurrent glioblastoma | calorie-restricted ketogenic diet | 3 days | 3 days | 250–485 days | N/A | Gastrointestinal symptoms, headache, muscle  cramps |
| **Phillips et al., 2022**  (38) | New zealand | Prospective Case Series | 10 | 58.0 +/−11.9 (range, 40–74) | glioblastoma multiforme | modified ketogenic diet | 127 ± 59 (79%) days | N/A | Median: 13 Months | N/A | Fatigue, irritability, and feeling lightheaded. No  Grade 3 or higher adverse events. |
| **Schwartz et al., 2022(39)** | USA | Prostective Study | 12 | N/A | glioblastoma multiforme | Ketogenic Diet | 6 weeks | N/A | Not Reported | N/A | N/A |
| **Smith K 2022(40)** | USA | retrospective observational study. | 16 | 48.3 (4.8) years. | 8 grade IV gliomas (seven glioblastoma, one  gliosarcoma), 7 grade III gliomas (three oligodendroglioma, four astrocytoma), and 1  grade II oligodendroglioma. IDH1 mutation status was present in 12 patients | KMT | 20.6 (13.8) months. | N/A | N/A | 20.0 (14.4) months. | No Adverse events |
| **Philli s et al., 2024**  (41) | New zealand | Case Report | 1 | 64-year | IDH-wildtype GBM | time-restricted ketogenic diet | 2 year | 2 year | 36 Months | N/A | Prolonged fasts caused mild fatigue, diarrhea, and  cold intolerance. No adverse events for KD. |
| **Laguado MIZ 2024**  (42) | Columbia | Observational multicentre study | 29 | 45 | glioblastoma  multiforme (n=15), grade 3 astrocytoma (n=6), grade 3 oligodendroglioma (n=5), and grade 3 pilocytic astrocytoma (n=3), | KMT + liquid ketogenic formula | 12 month | 12-month | N/A | N/A | acute diarrhea (17.2% - n=5), followed by grade 1 vomiting (10.3% - n=3) and grade 1 constipation |
| **Kiryttopoulos A 2025**  (8) | USA | prospective study | 18 | (median age 57.5 years) | diagnosed glioblastoma  multiforme | Ketogenic diet | 6 months | 6 months | 3-year survival rate is4/6 = 66.7%. | N/A | N/A |
| **J.Amaral 2025**  (7) | Cedars-Sinai Medical Center | open-  label study | 20 | 55 (26–71) | glioblastoma | KD diet+temozolomide chemotherapy | 16-week | 16 week | 29.4 months. | 12.9 months | No Adverse events |
| **William M**  (43) | UK | Conference abstract | Planned (Not specified) | 18+ | High-Grade Gliomas (HGG) & Low-Grade Gliomas (LGG) | MKD + Standard Chemoradiotherapy (HGG) or standard care (LGG) | Not specified (Duration of the trial) | Not specified (Duration of the trial) | Data not yet available (Trial not started) | Data not yet available (Trial not started) | Data not yet available (Trial not started). |
| **Shen 2016**  (44) | USA | Conference abstract | 1 | 7 | GBM | Ketogenic Diet as a Complementary Therapy during Chemoradiation | 4 months (initiated at the start of chemoradiation, with follow-up at 10 and 16 weeks) | At least 4 months (Data reported at 10 and 16 weeks post-KD initiation) | Not explicitly stated. | Not explicitly stated. | Mild decrease in serum carnitine; no other side effects noted. |
| **Dardis C 2017**  (45) | USA | Conference abstract | 14 | Not specified | Newly Diagnosed Glioblastoma | Classic 4:1 KD during RT + TMZ, then encouraged to follow Modified Atkins Diet (1:1) | Intensive phase: 1.5 months (duration of RT) | Not specified | Intention-to-treat (n=14): Median TTP: 7.0; Median OS: 29.3  Total on regimen (n=20): Median TTP: 6.6; Median OS: 22.0 | Not available. | - 2/14 patients stopped treatment due to tolerability.  - No weight loss >10% from baseline.  - Nausea was reported in 4/14 patients.  - No other significant adverse events. |
| **Gresham G 2019**  (46) | USA | Conference abstract | 20 | Not Specified | Newly diagnosed Glioblastoma | 16-week Ketogenic Diet + Standard-of-Care treatment | 4 months (Planned for all patients) | Not Yet Available (Study ongoing) | Not Yet Available (Study ongoing) | Not Yet Available (Study ongoing) | No Adverse events (accrual is ongoing) |
| **Schwartz K 2019**  (47) | USA | Conference abstract | 9 | Mean: 45 | GBM | Ketogenic Diet + RT + TMZ | 1.5 months (6 weeks) | Up to 49 months | Younger pts (n=3, mean age 28): No progression at 49, 31, and 32 mos. Older pts (n=6, mean age 54): Died at 25, 25, 13, 12, 9, and 9 mos. | N/A | No Adverse events |
| **N. Shinojima**  **(48)** | Japan | Conference abstract | 1 | 37 | GBM (progressive) | Palliative care with Ketogenic Diet | ≥8 (MRI showed response at 8 months) | 20 months (from diagnosis) | 20 months | N/A | No Adverse events |
| **S.Smiti**  (49) | Morocco | Conference abstract | 10 | Not Reported | GBM | Ketogenic Diet during radiotherapy vs. Usual Diet | 3 months | 3 months | Not Reported | N/A | N/A |
| **Nelson T 2021(50)** | USA | Conference abstract | 14 | Median: 55 | Newly diagnosed Glioblastoma (GBM) | KD+ Standard-of-Care | 4 months (16 weeks) | Up to 4 months (16 weeks) | 14.6 mo | N/A | No Adverse  events |

**Supplementary Table 4:** Joanna Briggs Institute (JBI) Critical Appraisal Checklist

**Nebeling et al., 1995**

| **Item** | **Question** | **Judgment** |
| --- | --- | --- |
| 1 | Were patient demographic characteristics clearly described? | Yes |
| 2 | Was the patient’s history clearly described and presented as a timeline? | Yes |
| 3 | Was the current clinical condition of the patient clearly described? | Yes |
| 4 | Were diagnostic tests or assessment methods clearly described? | Yes |
| 5 | Was the intervention(s) clearly described? | Yes |
| 6 | Was the post-intervention clinical condition clearly described? | Yes |
| 7 | Were adverse events or unanticipated events identified and described? | Yes |
| 8 | Does the case report provide takeaway lessons? | Yes |
| 9 | **JBI Appraisal Score** | **8 / 8** |
| 10 | **Overall Methodological Quality** | **High (100%)** |

**Zuccoli et al., 2010**

| **Sr No.** | **Criterion** | **Zuccoli et al., 2010** |
| --- | --- | --- |
| 1 | Were patient’s demographic characteristics clearly described? | **Yes** |
| 2 | Was the patient’s history clearly described and presented as a timeline? | **Yes** |
| 3 | Was the current clinical condition of the patient on presentation clearly described? | **Yes** |
| 4 | Were diagnostic tests or assessment methods and the results clearly described? | **Yes** |
| 5 | Was the intervention(s) or treatment procedure(s) clearly described? | **Yes** |
| 6 | Was the post-intervention clinical condition clearly described? | **Yes** |
| 7 | Were adverse events (harms) or unanticipated events identified and described? | **Yes** |
| 8 | Does the case report provide takeaway lessons? | **Yes** |
| 9 | **JBI Appraisal Score** | **8 / 8** |
| 10 | **Overall Methodological Quality** | **High (100%)** |

**Han et al., 2014**

| **Item** | **Question** | **Judgment** |
| --- | --- | --- |
| 1 | Were the two groups similar and recruited from the same population? | Yes |
| 2 | Were the exposures measured similarly to assign people to groups? | Yes |
| 3 | Was the exposure measured in a valid and reliable way? | Yes |
| 4 | Were confounding factors identified? | Yes |
| 5 | Were strategies to deal with confounding factors stated? | No |
| 6 | Were participants free of the outcome at the start of the study? | Yes |
| 7 | Were outcomes measured in a valid and reliable way? | Yes |
| 8 | Was follow-up time reported and sufficient? | Yes |
| 9 | Was follow-up complete and adequately described? | Yes |
| 10 | Were strategies to address incomplete follow-up utilized? | Unclear |
| 11 | Was appropriate statistical analysis used? | Yes |
| 12 | **JBI Appraisal Score** | **9 / 11** |
| 13 | **Overall Methodological Quality** | **High (82%)** |

**Rieger et al., 2014**

| **Sr No.** | **Criterion** | **Judgment** |
| --- | --- | --- |
| 1 | Were there clear criteria for inclusion in the case series? | Yes |
| 2 | Was the condition measured in a standard, reliable way? | Yes |
| 3 | Were valid methods used for identification of the condition? | Yes |
| 4 | Did the case series have consecutive inclusion of participants? | Yes |
| 5 | Did the case series have complete inclusion of participants? | No |
| 6 | Was there clear reporting of participant demographics? | Yes |
| 7 | Was there clear reporting of clinical information? | Yes |
| 8 | Were outcomes or follow-up results clearly reported? | Yes |
| 9 | Was there clear reporting of the presenting site(s)/clinic(s)? | Yes |
| 10 | Was statistical analysis appropriate? | Yes |
| 11 | **JBI Appraisal Score** | **9 / 10** |
| 12 | **Overall Methodological Quality** | **High (90%)** |

**Champ et al., 2014**

| **Sr No** | **Criterion** | **Champ et al., 2014** |
| --- | --- | --- |
| 1 | Were the two groups similar and recruited from the same population? | Yes |
| 2 | Were the exposures measured similarly to assign people to both exposed and unexposed groups? | Yes |
| 3 | Was the exposure measured in a valid and reliable way? | Yes |
| 4 | Were confounding factors identified? | Yes |
| 5 | Were strategies to deal with confounding factors stated? | No |
| 6 | Were the groups/participants free of the outcome at the start of the study? | Yes |
| 7 | Were the outcomes measured in a valid and reliable way? | Yes |
| 8 | Was the follow-up time reported and sufficient for outcomes to occur? | Yes |
| 9 | Was follow-up complete, and if not, were reasons for loss to follow-up described? | Yes |
| 10 | Were strategies to address incomplete follow-up utilized? | Yes |
| 11 | Was appropriate statistical analysis used? | Yes |
| 12 | **JBI Appraisal Score** | **10 / 11** |
| 13 | **Overall Methodological Quality** | **High (91%)** |

**Schwartz et al., 2022**

| **Sr No.** | **Criterion** | **Judgment** |
| --- | --- | --- |
| 1 | Were there clear criteria for inclusion in the case series? | Yes |
| 2 | Was the condition measured in a standard, reliable way? | Yes |
| 3 | Were valid methods used for identification of the condition? | Yes |
| 4 | Did the case series have consecutive inclusion of participants? | Unclear |
| 5 | Did the case series have complete inclusion of participants? | No |
| 6 | Was there clear reporting of participant demographics? | Yes |
| 7 | Was there clear reporting of clinical information? | Yes |
| 8 | Were outcomes or follow-up results clearly reported? | Yes |
| 9 | Was there clear reporting of the presenting site(s)/clinic(s)? | Yes |
| 10 | Was statistical analysis appropriate? | Yes |
| 11 | **JBI Appraisal Score** | **8 / 10** |
| 12 | **Overall Methodological Quality** | **High (80%)** |

**Artzi et al., 2017**

| **Item** | **Criterion** | **Judgment** |
| --- | --- | --- |
| 1 | Clear inclusion criteria? | Yes |
| 2 | Condition measured reliably? | Yes |
| 3 | Valid identification methods? | Yes |
| 4 | Consecutive inclusion? | Unclear |
| 5 | Complete inclusion? | No |
| 6 | Demographics reported? | Yes |
| 7 | Clinical info reported? | Yes |
| 8 | Outcomes reported? | Yes |
| 9 | Setting reported? | Yes |
| 10 | Appropriate statistics? | Yes |
| 11 | **JBI Score** | **8 / 10** |
| 12 | **Overall Quality** | **High (80%)** |

**Santos et al 2017**

| **Item** | **Question** | **Judgment** |
| --- | --- | --- |
| 1 | Were the two groups similar and recruited from the same population? | Yes |
| 2 | Were the exposures measured similarly to assign people to groups? | Yes |
| 3 | Was the exposure measured in a valid and reliable way? | Yes |
| 4 | Were confounding factors identified? | Yes |
| 5 | Were strategies to deal with confounding factors stated? | No |
| 6 | Were participants free of the outcome at the start of the study? | Yes |
| 7 | Were outcomes measured in a valid and reliable way? | Yes |
| 8 | Was follow-up time reported and sufficient? | Yes |
| 9 | Was follow-up complete and adequately described? | Yes |
| 10 | Were strategies to address incomplete follow-up utilized? | Unclear |
| 11 | Was appropriate statistical analysis used? | Yes |
| 12 | **JBI Appraisal Score** | **9 / 11** |
| 13 | **Overall Methodological Quality** | **High (82%)** |

**van der Louw et al., 2018**

| **Sr No.** | **Criterion** | **Judgment** |
| --- | --- | --- |
| 1 | Clear inclusion criteria? | Yes |
| 2 | Condition measured reliably for all participants? | Yes |
| 3 | Valid identification methods used? | Yes |
| 4 | Consecutive inclusion of participants? | No |
| 5 | Complete inclusion of participants? | No |
| 6 | Participant demographics clearly reported? | Yes |
| 7 | Clinical information clearly reported? | Yes |
| 8 | Outcomes or follow-up clearly reported? | Yes |
| 9 | Presenting site(s)/clinic(s) clearly reported? | Yes |
| 10 | Statistical analysis appropriate? | Yes |
| 11 | **JBI Appraisal Score** | **8 / 10** |
| 12 | **Overall Methodological Quality** | **High (80%)** |

**Martin-McGill et al., 2018**

| **Sr No.** | **Criterion** | **Judgment** |
| --- | --- | --- |
| 1 | Were there clear criteria for inclusion in the case series? | Yes |
| 2 | Was the condition measured in a standard, reliable way for all participants? | Yes |
| 3 | Were valid methods used for identification of the condition? | Yes |
| 4 | Did the case series have consecutive inclusion of participants? | Unclear |
| 5 | Did the case series have complete inclusion of participants? | No |
| 6 | Was there clear reporting of participant demographics? | Yes |
| 7 | Was there clear reporting of clinical information? | Yes |
| 8 | Were outcomes or follow-up results clearly reported? | Yes |
| 9 | Was there clear reporting of the presenting site(s)/clinic(s)? | Yes |
| 10 | Was statistical analysis appropriate? | Yes |
| 11 | **JBI Appraisal Score** | **8 / 10** |
| 12 | **Overall Methodological Quality** | **High (80%)** |

**Elsakka et al., 2018**

| **Item** | **Criterion** | **Judgment** |
| --- | --- | --- |
| 1 | Demographics described? | Yes |
| 2 | Timeline/history clear? | Yes |
| 3 | Clinical condition described? | Yes |
| 4 | Diagnostics described? | Yes |
| 5 | Intervention described? | Yes |
| 6 | Post-intervention status? | Yes |
| 7 | Adverse events reported? | Yes |
| 8 | Takeaway lessons? | Yes |
| 9 | **JBI Score** | **8 / 8** |
| 10 | **Overall Quality** | **High (100%)** |

**van der Louw et al., 2019**

| **Sr No.** | **Criterion** | **judgement** |
| --- | --- | --- |
| 1 | Were there clear criteria for inclusion in the case series? | Yes |
| 2 | Was the condition measured in a standard, reliable way for all participants included in the case series? | Yes |
| 3 | Were valid methods used for identification of the condition for all participants included in the case series? | Yes |
| 4 | Did the case series have consecutive inclusion of participants? | Unclear |
| 5 | Did the case series have complete inclusion of participants? | No |
| 6 | Was there clear reporting of the demographics of the participants in the study? | Yes |
| 7 | Was there clear reporting of clinical information of the participants? | Yes |
| 8 | Were the outcomes or follow up results of cases clearly reported? | Yes |
| 9 | Was there clear reporting of the presenting site(s)/clinic(s) demographic information? | Yes |
| 10 | Was statistical analysis appropriate? | Yes |
| 11 | **JBI Appraisal Score** | **8 / 10** |
| 12 | **Overall Methodological Quality** | **High (80%)** |

**Woodhouse et al., 2019**

| **Item** | **Criterion** | **Judgment** |
| --- | --- | --- |
| 1 | Clear inclusion criteria? | Yes |
| 2 | Condition measured reliably? | Yes |
| 3 | Valid identification methods? | Yes |
| 4 | Consecutive inclusion? | Yes |
| 5 | Complete inclusion? | Yes |
| 6 | Demographics reported? | Yes |
| 7 | Clinical info reported? | Yes |
| 8 | Outcomes reported? | Yes |
| 9 | Setting reported? | Yes |
| 10 | Appropriate statistics? | Yes |
| 11 | **JBI Score** | **10 / 10** |
| 12 | **Overall Quality** | **High (100%)** |

**Berrington et al., 2019**

| **Item** | **Criterion** | **Judgment** |
| --- | --- | --- |
| 1 | Clear inclusion criteria? | Yes |
| 2 | Condition measured reliably? | Yes |
| 3 | Valid identification methods? | Yes |
| 4 | Consecutive inclusion? | Yes |
| 5 | Complete inclusion? | Yes |
| 6 | Demographics reported? | Yes |
| 7 | Clinical info reported? | Yes |
| 8 | Outcomes reported? | Yes |
| 9 | Setting reported? | Yes |
| 10 | Appropriate statistics? | Yes |
| 11 | **JBI Score** | **10 / 10** |
| 12 | **Overall Quality** | **High (100%)** |

**Tóth et al., 2019**

| **Item** | **Criterion** | **Judgment** |
| --- | --- | --- |
| 1 | Demographics described? | Yes |
| 2 | Timeline/history clear? | Yes |
| 3 | Clinical condition described? | Yes |
| 4 | Diagnostics described? | Yes |
| 5 | Intervention described? | Yes |
| 6 | Post-intervention status? | Yes |
| 7 | Adverse events reported? | Yes |
| 8 | Takeaway lessons? | Yes |
| 9 | **JBI Score** | **8 / 8** |
| 10 | **Overall Quality** | **High (100%)** *(methodological; evidence level remains very low)* |

**Martin-McGill et al., 2020**

| **Sr No.** | **Criterion** | **Judgment** |
| --- | --- | --- |
| 1 | True randomization used? | Yes |
| 2 | Allocation to groups concealed? | Yes |
| 3 | Groups similar at baseline? | Yes |
| 4 | Participants blinded? | No |
| 5 | Treatment providers blinded? | No |
| 6 | Outcome assessors blinded? | Unclear |
| 7 | Groups treated identically other than intervention? | Yes |
| 8 | Follow-up complete and adequately described? | Yes |
| 9 | Participants analyzed in randomized groups? | Yes |
| 10 | Outcomes measured similarly for groups? | Yes |
| 11 | Outcomes measured reliably? | Yes |
| 12 | Appropriate statistical analysis used? | Yes |
| 13 | Trial design appropriate and deviations addressed? | Yes |
| 14 | **JBI Appraisal Score** | **11 / 13** |
| 15 | **Overall Methodological Quality** | **High (85%)** |

**Klein et al., 2020**

| **Sr No.** | **Criterion** | **Judgment** |
| --- | --- | --- |
| 1 | Clear inclusion criteria? | Yes |
| 2 | Condition measured reliably for all participants? | Yes |
| 3 | Valid methods used for condition identification? | Yes |
| 4 | Consecutive inclusion of participants? | Unclear |
| 5 | Complete inclusion of participants? | No |
| 6 | Demographics clearly reported? | Yes |
| 7 | Clinical information clearly reported? | Yes |
| 8 | Outcomes/follow-up clearly reported? | Yes |
| 9 | Presenting site(s)/clinic(s) clearly reported? | Yes |
| 10 | Appropriate statistical analysis used? | Yes |
| 11 | **JBI Appraisal Score** | **8 / 10** |
| 12 | **Overall Methodological Quality** | **High (80%)** |

**Panhans et al., 2020**

| **Sr No.** | **Criterion** | **Judgment** |
| --- | --- | --- |
| 1 | Were there clear criteria for inclusion in the case series? | Yes |
| 2 | Was the condition measured in a standard, reliable way? | Yes |
| 3 | Were valid methods used for identification of the condition? | Yes |
| 4 | Did the case series have consecutive inclusion of participants? | Unclear |
| 5 | Did the case series have complete inclusion of participants? | No |
| 6 | Was there clear reporting of participant demographics? | Yes |
| 7 | Was there clear reporting of clinical information? | Yes |
| 8 | Were outcomes or follow-up results clearly reported? | Yes |
| 9 | Was there clear reporting of the presenting site(s)/clinic(s)? | Yes |
| 10 | Was statistical analysis appropriate? | Yes |
| 11 | **JBI Appraisal Score** | **8 / 10** |
| 12 | **Overall Methodological Quality** | **High (80%)** |

**Voss et al., 2020**

| **Sr No.** | **Criterion** | **Judgment** |
| --- | --- | --- |
| 1 | True randomization used? | Yes |
| 2 | Allocation to groups concealed? | Yes |
| 3 | Groups similar at baseline? | Yes |
| 4 | Participants blinded? | No |
| 5 | Treatment providers blinded? | No |
| 6 | Outcome assessors blinded? | Yes |
| 7 | Groups treated identically other than intervention? | Yes |
| 8 | Follow-up complete and adequately described? | Yes |
| 9 | Participants analyzed in randomized groups? | Yes |
| 10 | Outcomes measured similarly for groups? | Yes |
| 11 | Outcomes measured reliably? | Yes |
| 12 | Appropriate statistical analysis used? | Yes |
| 13 | Trial design appropriate and deviations addressed? | Yes |
| 14 | **JBI Appraisal Score** | **11 / 13** |
| 15 | **Overall Methodological Quality** | **High (85%)** |

**Wenger et al., 2020**

| **Item** | **Criterion** | **Judgment** |
| --- | --- | --- |
| 1 | True randomization? | Yes |
| 2 | Allocation concealed? | Yes |
| 3 | Baseline similarity? | Yes |
| 4 | Participant blinding? | No |
| 5 | Provider blinding? | No |
| 6 | Outcome assessor blinding? | Yes |
| 7 | Identical treatment except intervention? | Yes |
| 8 | Follow-up adequate? | Yes |
| 9 | Intention-to-treat? | Yes |
| 10 | Outcome measurement consistent? | Yes |
| 11 | Outcome measurement reliable? | Yes |
| 12 | Appropriate statistics? | Yes |
| 13 | Trial design appropriate? | Yes |
| 14 | **JBI Score** | **11 / 13** |
| 15 | **Overall Quality** | **High (85%)** |

**Foppiani et al., 2020**

| **Item** | **Criterion** | **Judgment** |
| --- | --- | --- |
| 1 | Clear inclusion criteria? | Yes |
| 2 | Condition measured reliably? | Yes |
| 3 | Valid identification methods? | Yes |
| 4 | Consecutive inclusion? | Yes |
| 5 | Complete inclusion? | Yes |
| 6 | Demographics reported? | Yes |
| 7 | Clinical info reported? | Yes |
| 8 | Outcomes reported? | Yes |
| 9 | Setting reported? | Yes |
| 10 | Appropriate statistics? | Yes |
| 11 | **JBI Score** | **10 / 10** |
| 12 | **Overall Quality** | **High (100%)** |

**Schreck et al., 2019**

| **Item** | **Question** | **Judgment** |
| --- | --- | --- |
| 1 | Were there clear criteria for inclusion in the case series? | Yes |
| 2 | Was the condition measured in a standard, reliable way for all participants? | Yes |
| 3 | Were valid methods used for identification of the condition? | Yes |
| 4 | Did the case series have consecutive inclusion of participants? | Yes |
| 5 | Did the case series have complete inclusion of participants? | No |
| 6 | Was there clear reporting of participant demographics? | Yes |
| 7 | Was there clear reporting of clinical information? | Yes |
| 8 | Were outcomes or follow-up results clearly reported? | Yes |
| 9 | Was there clear reporting of the presenting site(s)/clinic(s)? | Yes |
| 10 | Was statistical analysis appropriate? | Yes |
| 11 | **JBI Appraisal Score** | **9 / 10** |
| 12 | **Overall Methodological Quality** | **High (90%)** |

**Perez et al., 2021**

| **Sr No.** | **Criterion** | **Perez et al., 2021** |
| --- | --- | --- |
| 1 | Were there clear criteria for inclusion in the case series? | Yes |
| 2 | Was the condition measured in a standard, reliable way for all participants? | Yes |
| 3 | Were valid methods used for identification of the condition? | Yes |
| 4 | Did the case series have consecutive inclusion of participants? | No |
| 5 | Did the case series have complete inclusion of participants? | Yes |
| 6 | Was there clear reporting of participant demographics? | Yes |
| 7 | Was there clear reporting of clinical information? | Yes |
| 8 | Were outcomes or follow-up results clearly reported? | Yes |
| 9 | Was there clear reporting of the presenting site(s)/clinic(s)? | Yes |
| 10 | Was statistical analysis appropriate? | Yes |
| 11 | **JBI Appraisal Score** | **9 / 10** |
| 12 | **Overall Methodological Quality** | **High (90%)** |

**Seyfried et al., 2021**

| **Sr No.** | **Criterion** | **Judgment** |
| --- | --- | --- |
| 1 | Were patient demographic characteristics clearly described? | Yes |
| 2 | Was the patient’s history clearly described as a timeline? | Yes |
| 3 | Was the current clinical condition clearly described? | Yes |
| 4 | Were diagnostic tests and results clearly described? | Yes |
| 5 | Was the intervention(s) clearly described? | Yes |
| 6 | Was the post-intervention clinical condition clearly described? | Yes |
| 7 | Were adverse or unanticipated events identified and described? | Yes |
| 8 | Does the case report provide takeaway lessons? | Yes |
| 9 | **JBI Appraisal Score** | **8 / 8** |
| 10 | **Overall Methodological Quality** | **High (100%)** |

**Proper et al 2021**

| **Sr No** | **Criterion** |  |
| --- | --- | --- |
| 1 | Were the two groups similar and recruited from the same population? | Yes |
| 2 | Were the exposures measured similarly to assign people to both exposed and unexposed groups? | Yes |
| 3 | Was the exposure measured in a valid and reliable way? | Yes |
| 4 | Were confounding factors identified? | Yes |
| 5 | Were strategies to deal with confounding factors stated? | No |
| 6 | Were the groups/participants free of the outcome at the start of the study? | Yes |
| 7 | Were the outcomes measured in a valid and reliable way? | Yes |
| 8 | Was the follow-up time reported and sufficient for outcomes to occur? | Yes |
| 9 | Was follow-up complete, and if not, were reasons for loss to follow-up described? | Yes |
| 10 | Were strategies to address incomplete follow-up utilized? | Yes |
| 11 | Was appropriate statistical analysis used? | Yes |
| 12 | **JBI Appraisal Score** | **10 / 11** |
| 13 | **Overall Methodological Quality** | **High (91%)** |

**Voss et al., 2022**

| **Sr No.** | **Criterion** | **Judgment** |
| --- | --- | --- |
| 1 | Was true randomization used for assignment of participants to treatment groups? | **Yes** |
| 2 | Was allocation to treatment groups concealed? | **Unclear** |
| 3 | Were treatment groups similar at the baseline? | **Yes** |
| 4 | Were participants blind to treatment assignment? | **No** |
| 5 | Were those delivering treatment blind to treatment assignment? | **No** |
| 6 | Were outcomes assessors blind to treatment assignment? | **Unclear** |
| 7 | Were treatment groups treated identically other than the intervention of interest? | **Yes** |
| 8 | Was follow up complete and if not, were differences between groups in terms of their follow up adequately described and analyzed? | **Yes** |
| 9 | Were participants analyzed in the groups to which they were randomized? | **Yes** |
| 10 | Were outcomes measured in the same way for treatment groups? | **Yes** |
| 11 | Were outcomes measured in a reliable way? | **Yes** |
| 12 | Was appropriate statistical analysis used? | **Yes** |
| 13 | Was the trial design appropriate, and any deviations from the standard RCT remember accounted for in the conduct and analysis of the trial? | **Yes** |
| 14 | **JBI Appraisal Score** | **10 / 13** |
| 15 | **Overall Methodological Quality** | **High (77%)** |

**Phillips et al., 2022**

| **Item** | **Question** | **Judgment** |
| --- | --- | --- |
| 1 | Were there clear criteria for inclusion in the case series? | Yes |
| 2 | Was the condition measured in a standard, reliable way for all participants? | Yes |
| 3 | Were valid methods used for identification of the condition? | Yes |
| 4 | Did the case series have consecutive inclusion of participants? | Yes |
| 5 | Did the case series have complete inclusion of participants? | Yes |
| 6 | Was there clear reporting of participant demographics? | Yes |
| 7 | Was there clear reporting of clinical information? | Yes |
| 8 | Were outcomes or follow-up results clearly reported? | Yes |
| 9 | Was there clear reporting of the presenting site(s)/clinic(s)? | Yes |
| 10 | Was statistical analysis appropriate? | Yes |
| 11 | **JBI Appraisal Score** | **10 / 10** |
| 12 | **Overall Methodological Quality** | **High (100%)** |

**Schwartz et al., 2022**

| **Sr No.** | **Criterion** | **Schwartz et al., 2022** |
| --- | --- | --- |
| 1 | Patient demographics clearly described? | Yes |
| 2 | Patient history presented as timeline? | Yes |
| 3 | Clinical condition on presentation clearly described? | Yes |
| 4 | Diagnostic tests and results clearly described? | Yes |
| 5 | Intervention(s) clearly described? | Yes |
| 6 | Post-intervention condition clearly described? | Yes |
| 7 | Adverse events identified and described? | Yes |
| 8 | Takeaway lessons provided? | Yes |
| 9 | **JBI Appraisal Score** | **8 / 8** |
| 10 | **Overall Methodological Quality** | **High (100%)** |

**Smith et al., 2022**

| **Item** | **Criterion** | **Judgment** |
| --- | --- | --- |
| 1 | Clear inclusion criteria? | Yes |
| 2 | Condition measured reliably? | Yes |
| 3 | Valid identification methods? | Yes |
| 4 | Consecutive inclusion? | Yes |
| 5 | Complete inclusion? | Yes |
| 6 | Demographics reported? | Yes |
| 7 | Clinical info reported? | Yes |
| 8 | Outcomes reported? | Yes |
| 9 | Setting reported? | Yes |
| 10 | Appropriate statistics? | Yes |
| 11 | **JBI Score** | **10 / 10** |
| 12 | **Overall Quality** | **High (100%)** |

**Phillips et al., 2024**

| **Sr No.** | **Criterion** | **Phillips et al., 2024** |
| --- | --- | --- |
| 1 | Were patient’s demographic characteristics clearly described? | Yes |
| 2 | Was the patient’s history clearly described and presented as a timeline? | Yes |
| 3 | Was the current clinical condition on presentation clearly described? | Yes |
| 4 | Were diagnostic tests or assessment methods and results clearly described? | Yes |
| 5 | Was the intervention(s) clearly described? | Yes |
| 6 | Was the post-intervention clinical condition clearly described? | Yes |
| 7 | Were adverse or unanticipated events identified and described? | Yes |
| 8 | Does the case report provide takeaway lessons? | Yes |
| 9 | **JBI Appraisal Score** | **8 / 8** |
| 10 | **Overall Methodological Quality** | **High (100%)** |

**Laguado et al., 2024**

| **Item** | **Criterion** | **Judgment** |
| --- | --- | --- |
| 1 | Clear inclusion criteria? | Yes |
| 2 | Condition measured reliably? | Yes |
| 3 | Valid identification methods? | Yes |
| 4 | Consecutive inclusion? | Yes |
| 5 | Complete inclusion? | Yes |
| 6 | Demographics reported? | Yes |
| 7 | Clinical info reported? | Yes |
| 8 | Outcomes reported? | Yes |
| 9 | Setting reported? | Yes |
| 10 | Appropriate statistics? | Yes |
| 11 | **JBI Score** | **10 / 10** |
| 12 | **Overall Quality** | **High (100%)** |

**Kiryttopoulos et al., 2025**

| **Item** | **Criterion** | **Judgment** |
| --- | --- | --- |
| 1 | Groups from same population? | Yes |
| 2 | Exposure measured similarly? | Yes |
| 3 | Exposure valid & reliable? | Yes |
| 4 | Confounders identified? | Yes |
| 5 | Confounders addressed? | No |
| 6 | Outcome-free at start? | Yes |
| 7 | Outcome measured reliably? | Yes |
| 8 | Follow-up sufficient? | Yes |
| 9 | Follow-up complete? | Yes |
| 10 | Incomplete follow-up addressed? | Unclear |
| 11 | Appropriate analysis? | Yes |
| 12 | **JBI Score** | **9 / 11** |
| 13 | **Overall Quality** | **High (82%)** |

**Amaral et al., 2025**

| **Item** | **Criterion** | **Judgment** |
| --- | --- | --- |
| 1 | Clear inclusion criteria? | Yes |
| 2 | Condition measured reliably? | Yes |
| 3 | Valid identification methods? | Yes |
| 4 | Consecutive inclusion? | Yes |
| 5 | Complete inclusion? | Yes |
| 6 | Demographics reported? | Yes |
| 7 | Clinical info reported? | Yes |
| 8 | Outcomes/follow-up reported? | Yes |
| 9 | Setting reported? | Yes |
| 10 | Appropriate statistics? | Yes |
| 11 | **JBI Score** | **10 / 10** |
| 12 | **Overall Quality** | **High (100%)** |
